# Supplementary material for: Low-Protein Formulas with Alpha-Lactalbumin-Enriched or Glycomacropeptide-Reduced Whey: Effects on Growth, Nutrient Intake and Protein Metabolism during Early Infancy: A Randomized, Double-Blinded Controlled Trial
Source: Nutrients. 2023 Feb 17;15(4):1010. doi: 10.3390/nu15041010 (PMC9958764; doi:10.3390/nu15041010)
Supplement: Supplementary file 1 [file nutrients-15-01010-s001.zip › supplementary-nutrients-2028454.pdf]

Supplementary Material

Low-Protein Formulas with Alpha-lactalbumin-enriched or Glycomacropeptide-reduced Whey: Effects on Growth, Nutrient Intake and Protein Metabolism during Early Infancy: A Randomized, Double-Blinded Controlled Trial

Ulrika Tinghäll Nilsson , Olle Hernell , Bo Lönnerdal , Merete Lindberg Hartvigsen , Lotte Neergaard Jacobsen , Anne Staudt Kvistgaard and Pia Karlsland Åkeson

Table S1. Nutrient content in study formulas and minimum levels according to regulation.

	**SF**[2]	**α-lac-EW**	**CGMP-RW**	**Regulation**[3]
Energy (kcal/100 ml)[1]	67.3	68.2	68.0	60
Whey: casein ratio	60:40	70:30	70:30	
Protein (g/100ml)	1.48	1.19	1.20	
Protein (g/ 100 kcal)	2.20	1.75	1.76	1.8
α-lactalbumin (%)	10	27	14	
Carbohydrate (g)	10.1	10.1	10.2	9
Fat (g)	5.6	5.7	5.7	4.4
Calcium (mg)	102.0	100.0	98.0	50
Phosphorous (mg)	57.1	63.6	64.1	25
Magnesium (mg)	10.0	10.0	10.0	5
Iron (mg)	1.2	1.2	1.1	0.3
Zinc (mg)	1.2	1.2	1.2	0.5
Manganese (μg)	54.6	53.6	53.1	1
Copper (μg)	67.1	69.0	59.7	35
Iodine (μg)	35.9	32.9	34.0	10
Sodium (mg)	27.0	27.0	29.0	20
Potassium (mg)	96.8	91.9	91.0	60
Chloride (mg)	70.4	71.0	69.0	50
Selenium (μg)	5.5	5.0	5.1	1
Vitamin A (μg ER)	163.2	171.0	166.7	60
Vitamin D3 (μg)	2.2	2.3	2.3	1
Vitamin E (mg αTE)	4.2	4.2	4.4	0.5
Vitamin K (μg)	19.4	20.6	20.8	4
Thiamine (B1) (μg)	257.9	253.5	252.2	60
Riboflavin (B2) (μg)	345.1	314.9	324.2	80
Niacin (B3) (μg)	1257.8	1270.0	1299.0	300
Pantothenic acid (B5) (μg)	1338.3	1256.0	1298.0	400
Pyridoxin (B6) (μg)	153.4	157.0	153.6	35
Biotin (B8) (μg)	6.3	5.9	6.0	1.5
Folic acid (B9) (μg)	44.9	41.6	42.0	10
Cobalamin (B12) (μg)	0.45	0.31	0.37	0.1
Vitamin C (mg)	23.0	23.8	23.5	10

[1]Energy in kcal/100 ml, protein content in g/100 ml and g/100 kcal, all other nutrients in /100 kcal. [2]SF, standard formula; α-lac-EW, experimental formula with α-lactalbumin-enriched whey; CGMP-RW, experimental formula with reduced CGMP whey. [3] EU Regulation [7].

Table S2. Amino acid content in study formulas and minimum levels according to regulation.

	SF[1]	α-lac-EW	CGMP-RW	Regulation[2]
Essential (mg/100 kcal)				
Cysteine	37	39	39	38
Histidine	56	42	45	40
Isoleucine	127	111	96	90
Leucine	225	160	201	166
Lysine	208	162	177	113
Methionine	53	36	42	23
Phenylalanine	89	65	72	83
Threonine	127	117	87	77
Tryptophan	37	36	35	32
Tyrosine	82	56	66	76
Valine	135	103	98	88
Nonessential (mg/100 kcal)				
Alanine	93	60	77	-
Arginine	65	45	51	-
Aspartic acids	211	186	182	-
Glutamic acid	439	314	344	-
Glycine	44	36	36	-
Proline	177	118	115	-
Serine	114	90	85	-

[1]SF, standard formula; α-lac-EW, experimental formula with α-lactalbumin-enriched whey; CGMP-RW, experimental formula with reduced CGMP whey.
[2] EU Regulation [7].

Table S3 a. Mean serum concentration of essential and nonessential amino acids at 2 months of age in a randomized subgroup of infants fed formula (SF, α-lac-EW or CGMP-RW)* or breast milk (BF).

	SF<br>*n*=50	α-lac-EW<br>*n*=50	CGMP-RW<br>*n*=50	BF<br>*n*=50
		2 mo		
Essential amino acids (µmol/L)				
Histidine	96.8[a] ± 14.2[1]	103.3[a,d] ± 21.1	93.6 ± 13.5	88.1 ± 11.7
Isoleucine	77.4[a] ± 14.6	75.9 ± 16.2	74.4 ± 17.9	68.0 ± 18.4
Leucine	136.4 ± 22.8	134.7 ± 25.7	131.5 ± 29.2	125.7 ± 29.6
Lysine	238.9[a] ± 45.2	242.1[a] ± 45.4	233.5[a] ± 39.0	184.8 ± 46.6
Methionine	33.0 ± 6.2	32.3 ± 7.8	30.2 ± 6.3	25.2 ± 4.9
Phenylalanine	68.1[a] ± 22.4	68.2[a] ± 11.5	66.9[a] ± 13.9	56.5 ± 11.1
Threonine	210.7[a] ± 43.6	211.4[a] ± 47.2	191.9[a] ± 43.2	140.6 ± 26.3
Tryptophan	76.8 ± 9.7	76.0 ± 12.1	74.4 ± 11.1	79.3 ± 12.8
Valine	196.9[a] ± 34.4	196.2[a] ± 41.1	192.2[a] ± 39.8	170.8 ± 33.9
BCAA**	410.1[a] ± 65.8	406.8[a] ± 77.5	398.0 ± 82.2	364.4 ± 78.1

Nonessential amino acids (μmol/L)				
Alanine	423.1 ± 74.0	433.9 ± 102.72	404.5 ± 84.52	395.5 ± 71.33
Arginine	109.3 ± 21.0	108.8 ± 24.5	111.0 ± 15.2	114.8 ± 24.3
Asparagine	59.3[a] ± 10.9	59.3[a] ± 15.7	56.30[a] ± 12.76	48.98 ± 12.96
Aspartic	27.96[b] ± 8.58	33.09[d] ± 11.95	28.28 ± 6.73	29.30 ± 9.46
Glutamic	204.9[a] ± 125.3	219.6[a] ± 83.34	216.7[a] ± 100.1	284.3 ± 159.3
Glutamine	450.6 ± 158.5	436.1 ± 147.2	464.1 ± 158.9	417.0 ± 180.4
Glycine	269.9[b] ± 46.9	305.9[a,d] ± 66.7	255.6 ± 42.3	254.7 ± 42.8
Proline	210.7[a] ± 37.6	211.5[a] ± 38.8	204.7[a] ± 36.0	246.3 ± 41.3
Serine	181.1[b] ± 49.5	218.4[d] ± 96.7	175.9 ± 39.4	192.1 ± 41.9
Tyrosine	101.7 ± 25.4	97.4 ± 25.2	96.8 ± 23.7	93.0 ± 19.6

[*]SF, standard formula; α-lac-EW, experimental formula with α-lactalbumin-enriched whey ; CGMP-RW, experimental formula with reduced CGMP whey. [1] Mean ± SD. Groups compared by one-way ANOVA, post hoc Bonferroni. P value < 0.05 are considered statistically significant and marked by superscript letters. [a] Significantly different vs. BF. [b] SF vs. α-lac-EW. [c] SF vs. CGMP-RW. [d] α-lac-EW vs. CGMP-RW. [**] Total mean of branched chain amino acids (BCAA), isoleucine, leucine and valine.

Table S3 b. Mean serum concentration of essential and nonessential amino acids at 4 months of age in a randomized subgroup of infants fed formula (SF, α-lac-EW or CGMP-RW)* or breastmilk (BF)

	**SF** *n*=50	**α-lac-EW** *n*=50	**CGMP-RW** *n*=50	**BF** *n*=50
		4 mo		
Essential amino acids (μmol/L)				
Histidine	74.8 ± 11.9[1]	72.3 ± 13.3	69.3[a] ± 9.6	78.5 ± 11.9
Isoleucine	90.5[a,c] ± 21.6	89.3[a,d] ± 22.7	68.5 ± 10.6	59.8 ± 15.1
Leucine	137.2[a,b] ± 31.0	106.7[d] ± 25.3	123.4[a] ± 31.4	98.7 ± 26.0
Lysine	213.5[a,b] ± 36.6	194.0[a] ± 36.0	198.8[a] ± 38.3	144.6 ± 28.3
Methionine	27.6[a,b] ± 6.4	23.0[a] ± 5.1	24.9[a] ± 6.4	19.0 ± 4.0
Phenylalanine	62.0[a] ± 8.9	55.1 ± 10.7	57.5 ± 10.2	52.0 ± 9 .3
Threonine	173.1[a,c] ± 32.8	216.6[a,d] ± 38.6	138.7 ± 31.1	126.1 ± 26.3
Tryptophan	68.4 ± 10.8	72.0 ± 11.9	68.5 ± 10.6	69.6 ± 11.6
Valine	229.0 [a,b,c] ± 38.6	196.8[a,d] ± 36.6	163.7 ± 32.4	149.4 ± 32.7
BCAA[**]	456.7 [a,b,c] ± 89.9	392.8[a] ± 83.0	357.0[a] ± 80.5	307.8 ± 72.3
Nonessential amino acids (μmol/L)				
Alanine	367.9 ± 66.7	408.3[a,b] ± 78.1	396.6 ± 86.1	370.6 ± 71.8
Arginine	109.7 ± 18.0	105.3 ± 16.4	108.7 ± 18.7	111.1 ± 16.2
Asparagine	59.0[a] ± 10.8	61.6[a,d] ± 13.2	55.2[a] ± 10.4	45.2 ± 8.8
Aspartic	26.4 ± 8.9	26.9 ± 8.9	26.0 ± 6.8	24.1 ± 7.1
Glutamic	167.2 ± 59.1	174.8 ± 56.3	186.8 ± 75.7	207.8 ± 102.1
Glutamine	442.0[a] ± 120.0	494.7 ± 117.1	479.3 ± 136.9	536.5 ± 166.6
Glycine	212.6 ± 43.0	243.1[b] ± 43.6	225.6 ± 40.3	229.1 ± 48.4
Proline	199.1[a,c] ± 35.1	185.1[a] ± 34.4	174.2[a] ± 27.9	218.9 ± 45.8

Serine	174.9 ± 53.1	191.4 ± 56.6	178.7 ± 44.1	184.9 ± 34.5
Tyrosine	90.6[a,b] ± 20.7	75.6 ± 18.6	83.3 ± 23.8	75.0 ± 16.5

*SF, standard formula; α-lac-EW, experimental formula with α-lactalbumin-enriched whey ; CGMP-RW, experimental formula with reduced CGMP whey.
[1] Mean ± SD. Groups compared by one-way ANOVA, post hoc Bonferroni.
P value < 0.05 are considered statistically significant and marked by superscript letters. [a] Significantly different vs. BF. [b] SF vs. α-lac-EW. [c] SF vs. CGMP-RW. [d] α-lac-EW vs. CGMP-RW. ** Total mean of branched chain amino acids (BCAA), isoleucine, leucine and valine.

Table S3 c. Mean serum concentration of essential and nonessential amino acids at 6 months of age in a randomized subgroup of infants fed formula (SF, α-lac-EW or CGMP-RW)* or breast milk (BF).

	**SF** *n*=50	**α-lac-EW** *n*=50	**CGMP-RW** *n*=50	**BF** *n*=50
		6 mo		
Essential amino acids (µmol/L)				
Histidine	71.3[a] ± 13.4[1]	65.3[a] ± 15.1	71.5[a] ± 13.8	80.8 ± 13.2
Isoleucine	90.9[a,c] ± 21.2	85.3[a,d] ± 20.3	73.8[a] ± 18.9	58.4 ± 13.1
Leucine	139.5[a,b] ± 31.4	107.4[d] ± 25.4	127.6[a] ± 35.9	93.8 ± 21.6
Lysine	199.2[a,b] ± 39.8	173.4[a] ± 43.0	188.4[a] ± 37.5	140.2 ± 28.3
Methionine	24.8[a,b] ± 7.1	20.3 ± 5.6	23.2[a] ± 5.4	18.2 ± 3.8
Phenylalanine	63.3[a,b] ± 9.5	58.6 ± 10.5	60.0 ± 11.8	54.8 ± 12.5
Threonine	153.8[a,b] ± 34.4	181.9[a,d] ± 49.0	139.2 ± 32.8	126.9 ± 25.8
Tryptophan	68.2 ± 9.2	68.3 ± 13.4	70.1[a] ± 12.5	63.3 ± 13.1
Valine	235.8[a,b,c] ± 39.3	197.6[a,d] ± 39.4	177.9[a] ± 41.1	157.0 ± 29.5
BCAA**	466.2[a,b,c] ± 90.4	388.3[a] ± 81.0	379.3[a] ± 90.1	309.2 ± 61.8
Nonessential amino acids (µmol/L)				
Alanine	353.2 ± 75.6	373.6 ± 90.0	400.4[c] ± 111.2	361.6 ± 73.6
Arginine	111.7 ± 18.9	102.8 ± 25.9	113.6 ± 22.7	112.1 ± 22.5
Asparagine	58.3[a] ± 12.2	56.7 [a] ± 16.0	57.2[a] ± 13.4	45.0 ± 11.5
Aspartic	27.0 ± 9.3	25.1 ± 8.5	27.3 ± 10.8	23.0 ± 6.5
Glutamic	167.2 ± 59.1	174.8 ± 56.3	186.8 ± 75.7	207.8 ± 102.1
Glutamine	467.1[a] ± 97.6	476.2[a] ± 100.6	506.3[a] ± 124.8	575.2 ± 137.5
Glycine	214.6 ± 37.3	240.4[b] ± 48.8	242.8[c] ± 50.8	225.2 ± 38.9
Proline	203.9[b,c] ± 42.6	182.5[a] ± 45.3	182.8[a] ± 35.9	220.2 ± 46.7
Serine	177.5 ± 37.9	177.1 ± 44.8	184.4 ± 55.7	189.4 ± 41.9
Tyrosine	87.4[a,b] ± 23.2	71.2 ± 19.1	83.3[a,c] ± 24.0	67.0 ± 14.0

*SF, standard formula; α-lac-EW, experimental formula with α-lactalbumin-enriched whey ; CGMP-RW, experimental formula with reduced CGMP whey.
[1] Mean ± SD. Groups compared by one-way ANOVA, post hoc Bonferroni.
P value < 0.05 are considered statistically significant and marked by superscript letters. [a] Significantly different vs. BF. [b] SF vs. α-lac-EW. [c] SF vs. CGMP-RW. [d] α-lac-EW vs. CGMP-RW. ** Total mean of branched chain amino acids (BCAA), isoleucine, leucine and valine.

Table S4. Gastrointestinal symptoms in infants fed formula (SF, α-lac-EW or CGMP-RW)* or breast milk (BF) during the intervention, ITT population.

	SF<br>*n* = 70	α-lac-EW<br>*n* = 73	CGMP-RW<br>*n* = 69	*P-value*[1]	BF<br>*n* = 71
Stool frequency[2]	1.3 ± 0.5[a,b,c]	1.1 ± 0.4[a]	1.1 ± 0.4[a]	0.001	2.0 ± 1.1
Watery stool[3]	1.0 (0;4)	1.4 (0;6.6)	0.6 (0;2.5)	0.18	0 (0;5.3)
Loose stools[3]	50.4 (27.2;79.4)[a]	49.1 (22;80.7)[a]	38.4 (15.8;75.5)[a]	0.26	96.8 (80.2;100)
Firm stools[3]	38.8 (8.7; 62.7)[a]	37 (7.2;73.3)[a]	45.8 (15.4;79.9)[a]	0.24	0 (0;7)
Hard stools[3]	0.1 (0;4.5)[a]	0 (0;4.2)[a]	1.0 (0;6.4)[a]	0.35	0 (0;0)
Stomach pain[4]	1.6 (0;6.3)	1.5 (0;8)	0.8 (0;4.5)	0.83	1.5 (0;3.5)
Vomiting[5]	2.2 (0;10)[a]	1.6 (0;11)[a]	1.4 (0;6.9)	0.62	0 (0;4.1)
Flatulence[6]	5.0 (0.7;25.7)	4.0 (0.6;16.6)	3.7 (0.6;10)	0.35	4.6(0;13.7)

*SF, standard formula; α-lac-EW, experimental formula with α-lactalbumin-enriched whey; CGMP-RW, experimental formula with reduced CGMP whey.
[1]Groups compared by one-way ANOVA, post hoc Bonferroni. Proportions compared by Kruskal-Wallis, post hoc Bonferroni. [2] Stools/day, mean ± SD.
[3] Proportion of defecations from parental registration in diary, median (25th;75th percentiles). [4]Proportion of days with stomach pain, median (25th;75th percentiles). [5] Proportion of days with vomiting, median (25th;75th percentiles).
[6] Proportion of days with flatulence, median (25th;75th percentiles). [a] Significantly different vs. BR ($p<0.05$). [b] SF vs. α-lac-EW. [c] SF vs. CGMP-RW.

Table S5. Estimated time to fall asleep, duration of crying and crying related to feeding as reported for 2 days/ week in infants fed formula (SF, α-lac-EW or CGMP-RW)* or breast milk (BF) during the intervention, ITT population.

	SF<br>*n*=70	α-lac-EW<br>*n*=73	CGMP-RW<br>*n*=68	*P-value*[1]	BF<br>*n*=70
Crying related to feeding[2]	1.25 (0;10.3)	0 (0;11.4)	0 (0;9)	0.77	0 (0;11.8)
Crying <1 h/d[3]	100 (93.0;100)	100 (94.4;100)	97.4 (94;100)	0.83	100 (95;100)
Crying <30 min/d[3]	85 (66.4;98.0)	87 (68;100)	86 (66.0;96.0)	0.65	89 (64.2;100.0)
Time to sleep < 5 min[4]	2.9 (0;32.7)	11 (0;40.0)	9 (0;36.0)	0.49	13.6 (0;34)
Time to sleep 5 to < 15 min[4]	39.4 (16.4,64.8)	41 (21.8,61.8)	50 (12.5;68.8)	0.71	46 (21.1;62.9)
Time to sleep 15 to < 30 min[4]	23.7 (10.0;44.4)	15.8 (5.5;35.7)	19.4 (2.9;35.2)	0.13	16.7 (3.4;39.2)
Time to sleep 30 to < 60 min[4]	3.3 (0;13.3)	2.8 (0;8.6)	0 (0;8.1)	0.27	2.5 (0;13.3)
Time to sleep > 60 min[4]	0 (0;2.6)	0 (0;2.7)	0 (0;0)	0.52	0 (0;0)

*SF, standard formula; α-lac-EW, experimental formula with α-lactalbumin-enriched whey; CGMP-RW, experimental formula with reduced CGMP whey.
[1] Proportions compared by Kruskal Wallis. [2] Proportion of days with crying during or after feeding, median (25th;75th percentiles). [3] Proportion of days with crying (25th;75th percentiles). [4] Proportion of days with time to fall asleep (25th;75th percentiles).
